# Determination of adipose tissue beiging from passive electrical and optical properties

**DOI:** 10.3389/fphys.2026.1917194

**Published:** 2026-10-29

**Authors:** Jie Hou, Florian Dapsance, Damien Dufour, Charlotte N. Boccara, Nolwenn Briand, Ørjan G. Martinsen

**Affiliations:** 1 Department of Physics, University of Oslo, Oslo, Norway; 2 Norwegian Centre for Molecular Biosciences and Medicine, University of Oslo, Oslo, Norway; 3 Department of Molecular Medicine, Institute of Basic Medical Sciences, University of Oslo, Oslo, Norway; 4 Department of Neurology, Oslo University Hospital, Oslo, Norway; 5 Department of Clinical and Biomedical Engineering, Oslo University Hospital, Oslo, Norway

**Keywords:** adipose tissue, dielectric relaxation spectroscopy, electrical impedance spectroscopy, machine learning, near-infrared (NIR) spectroscopy

## Abstract

**Introduction:**

Adipose tissue plays a central role in mammalian energy homeostasis and metabolic regulation, and this study aims to characterize functionally distinct adipose depots and their dynamic adaptations.

**Methods:**

Electrical Impedance Spectroscopy (EIS), Dielectric Relaxation Spectroscopy (DRS), and Near-Infrared Spectroscopy (NIRS) were used to analyze different types of adipose tissue, which were then classified using machine learning (ML), alongside extraction and validation of dielectric properties to estimate water content.

**Results:**

The results show that while inguinal white adipose tissue (iWAT) is morphologically closer to peri-gonadal white adipose tissue (gWAT), iWAT and brown adipose tissue (BAT) share more similar electrical and optical properties; moreover, ML methods applied to DRS measurements achieved excellent classification accuracy ($>99%$), and dielectric analysis enabled reliable estimation of depot-specific water content.

**Discussion:**

This approach provides a promising non-destructive method for studying adipose tissue adaptations and remodeling, including monitoring of beiging and the transdifferentiation of white adipocytes into highly metabolic beige adipocytes.

## 1 Introduction

Adipose tissue plays an important role in the energy balance of mammals by serving as a reserve source of energy. Standard histological methods based on chemical fixation can reveal the structure and function of adipose tissue, but sample preparation is time-consuming, resource-intensive, and destructive. Therefore, there is a need for alternative techniques to characterize adipose tissue while preserving its integrity – in a manner better suited for clinical and research applications.

Adipose tissue is distributed in several depots throughout the body and is broadly classified as white or brown (Sakers et al., 2022). Adipocytes are the functional units of adipose tissue and can display different morphologies and functions (Rodrigues et al., 2016). White adipocytes contain a single large lipid droplet that is used to store triglycerides. In contrast, brown and beige adipocytes contain multiple lipid droplets and many mitochondria, allowing them to generate heat and act as metabolic sinks in response to cold exposure or sympathetic nervous system activation (Li et al., 2022). These adipose depots also differ markedly in their physiological roles. White adipose tissue (WAT) primarily functions as an energy reservoir, releasing adipokines that regulate systemic metabolism. In contrast, brown adipose tissue (BAT) is specialized for non-shivering thermogenesis through uncoupled mitochondrial respiration. Beige adipocytes, which emerge within WAT depots such as inguinal white adipose tissue (iWAT), exhibit inducible thermogenic capacity and contribute to metabolic flexibility. These functional differences are associated with variations in vascularization, mitochondrial density, and lipid composition, all of which are expected to influence the electrical and optical properties measured in this study (Cedikova et al., 2016; Leiria and Tseng, 2020; Richard et al., 2020). These morphological and structural differences are expected to influence both electrical conduction pathways and optical absorption – and as such can be used to characterize different adipose tissue.

Brown adipose tissue (BAT) is located in the subcutaneous interscapular region and is abundant in mammals that rely on non-shivering thermogenesis to maintain body temperature (Cinti, 2005). In mice, the main WAT depots are visceral perigonadal WAT (gWAT) and subcutaneous inguinal WAT (iWAT). Unlike gWAT, iWAT can contain white and beige adipocytes, with beige cells appearing near the inguinal lymph node in response to cold exposure. As a result, iWAT can show features of both white and brown adipose tissue, making it a tractable model of adipose tissue plasticity for testing whether biophysical methods can detect partial beiging.

Based on known differences in cellular organization, lipid content, and water content among adipose tissue depots, we hypothesized that passive electrical and optical properties could provide complementary, non-destructive markers capable of distinguishing brown, beige-containing, and white adipose tissues. In particular, we expected that methods sensitive to membrane density, molecular polarization, and water–lipid balance would reflect functional differences that are not fully captured by histology alone.

Electrical impedance spectroscopy (EIS), also known as bioimpedance in biological applications, is a widely used technique for characterizing cells and tissues (Dean et al., 2008; Stupin et al., 2021; Martinsen and Heiskanen, 2023). It is based on the electrical conductivity of intra- and extracellular fluids and the capacitive properties of cell membranes. By applying an electrical field over a range of frequencies, EIS can provide information on cell size and density, membrane properties, and, to a limited extent, intracellular composition.

Dielectric relaxation spectroscopy (DRS) is a type of impedance spectroscopy that operates in the MHz and GHz frequency range. Because biological tissues have both conductive and insulating properties, they are considered dielectric materials, making DRS well-suited for their characterization. In addition, biological tissues can act as transmission channels for electromagnetic waves (Alyounes and Razan, 2022).

Near-infrared spectroscopy (NIRS) uses the interaction between light and matter to analyze the composition of organic tissues. When exposed to near-infrared light, molecules such as lipids and water exhibit characteristic absorption patterns. The amount of absorbed light is proportional to the concentration of each molecule, allowing both qualitative and quantitative tissue analysis.

Previous studies demonstrated the ability of each method, EIS (Gabriel et al., 2009; Kalvoy et al., 2009; Meroni et al., 2016; Dapsance et al., 2023; Khoumeri et al., 2026), DRS (Smith and Foster, 1985; Rodrigues et al., 2016), and NIRS (van Veen et al., 2004), to independently characterize the passive electrical and optical properties of adipose tissue. Together, these results highlight the low conductivity, low dielectric loss, and dielectric constant of adipose tissue due to its reduced water content. To date, there has been no report of combined spectroscopic approaches to reveal the physical properties of different types of adipose tissue.

In this study, we compared the electrical and near-infrared optical properties of three adipose tissue depots—gWAT, iWAT, and BAT—in a C57BL/6 mouse model. Each technique was chosen to investigate a distinct but overlapping biophysical aspect of adipose tissue: cellular structure (EIS), molecular polarization and water content (DRS), and lipid–water optical signatures (NIRS). Our results show that all three methods reveal similarities between iWAT and BAT, while gWAT exhibits distinct electrical and optical characteristics. Machine learning (ML) methods revealed that DRS outperformed EIS and NIRS in classifying adipose tissue. Finally, DRS-based water content estimates were validated against dynamic vapor sorption (DVS) to demonstrate how DRS can inform on the beiging state of iWAT – a crucial marker in metabolism research.

## 2 Methods

### 2.1 Animals and experimental design

Approval and registration for the experimental use of animals were granted by the Norwegian Animal Research Authority (Mattilsynet, KPM-IMB with FOTS ID 30147). Experiments were performed in accordance with the Norwegian Animal Welfare Act and the European Convention for the Protection of Vertebrate Animals used for Experimental and Other Scientific Purposes. Adult mice (aged between 18–35 weeks) of both sexes were bred in-house and kept on a C57BL/6J genetic background. They were housed in individually ventilated plexiglass cages with a 12-hour light/12-hour dark cycle (lights on at 7:00 am), given standard commercial rodent food ad libitum, and had unrestricted access to water. Cervical dislocation was employed for euthanasia between 9 and 10 am; all measurements were performed between 9:30 and 12:00 am. Cl-316, 243, or vehicle (phosphate-buffed saline) was administered by intraperitoneal injections (1 mg/kg) at 9 am on the two days before the experiments.

After termination, inguinal WAT and perigonadal WAT from each side, as well as interscapular BAT were collected. The number of depots used for each analysis is included in the figure captions. All measurements were initiated within minutes after tissue isolation to minimize post-mortem changes. Samples were temporarily stored in phosphate-buffered saline (PBS) at room temperature prior to measurement. No active perfusion or oxygenation was maintained, as the study focused on passive ex vivo properties. Tissue samples were used without normalization to mass or volume, but care was taken to ensure similar sample sizes and consistent electrode contact conditions across measurements.

### 2.2 Histological analysis

Tissues were fixed in 4% paraformaldehyde for 4–6 h and embedded in paraffin. 5μm sections were deparaffinized and processed for hematoxylin/eosin stainings. For PLIN1 immunofluorescence, deparaffinized slides were submerged in a citrate-based antigen retrieval buffer and microwaved for 8 min. After being rinsed with 1× PBS, slides were blocked for an hour with 2.5% horse serum (Vector) and incubated overnight at 4 °C with rabbit anti-PLIN1 antibody (Cell signaling technology #9349, 1/500). After rinsing, they were incubated with ImmPRESS polymer for 30 min at room temperature. HRP activity was detected with Alexafluor (Thermo Fisher). The images were acquired with Zeiss Axioscan Z1 and ZEN 3.4 blue edition software and analyzed with QuPath 0.4.2 software (Bankhead et al., 2017).

### 2.3 Electrical impedance spectroscopy

EIS measurements were performed using a custom-designed platform with 7-mm-wide Ag/AgCl strip electrodes arranged in a two-electrodee configuration and connected to a Zurich Instruments MFIA 5 MHz impedance analyzer, as previously described (Dapsance et al., 2023). Immediately after dissection, each tissue was placed in 1x PBS and gently pressed against a piece of gauze to remove excess liquid from its surface. A 100 mV peak sinusoidal voltage served as an excitation signal over a 10 Hz – 1 MHz frequency range.

Each impedance measurement can be expressed as Equation 1:

(1) $$\vec{Z}\;=R+jX$$

where Z is the complex impedance, R is the resistance, and X is the reactance. The real part R is typically associated with electrolytic intra- and extracellular conductivity/resistivity due to free ions and dielectric loss due to bound ions. The reactance X mainly results from capacitive effects at the cell membranes and potentially at the electrode/tissue interface.

In order to plot the impedance as a function of the frequency, the impedance module is obtained by Equation 2:

(2) $$\left|Z\right|=\sqrt{R^{2}+X^{2}}$$

The phase angle is a measure of the ratio between the out-of-phase component and the in-phase component. In the case of the impedance, the reactance, and the resistance, respectively. In other words, a higher phase angle suggests higher capacitive effects that typically originate from cell membranes. The phase angle is expressed by Equation 3:

(3) $$\varphi =tan^{-1}\left(\frac{X}{R}\right)$$

### 2.4 Dielectric relaxation spectroscopy

Dielectric relaxation spectroscopy (DRS) is a suitable method for studying the dielectric properties of dipolar molecules in the γ and δ dispersion range (Hou et al., 2022b, 2025; Hou et al., 2022a). DRS was performed using a vector network analyzer (VNA) alongside an open-ended coaxial probe (OCP) (Gregory and Clarke, 2007). Specifically, we used the DAK 3.5 probe covering frequencies from 200 MHz to 20 GHz (Schmid & Partner Engineering AG, Switzerland), in conjunction with the R140 VNA, which spans 85 MHz to 14 GHz (Copper Mountain Technologies, IN, USA). The combined frequency constraints of the OCP and VNA established the experimental frequency range from 200 MHz to 14 GHz.

Prior to each measurement, a three-point calibration, “Open”, “Short”, and “Load” (with deionized water) was conducted (Kaatze, 1989; Ellison, 2007). To verify the precision of the calibration, a 0.1 mol/L saline solution (Abdilla et al., 2013) was used, to ensure the accuracy of calibration by comparing the dielectric properties of saline with established reference data. The calibration was deemed satisfactory when the maximum difference observed between our measured data and the reference data for saline was within ±2%.

The extraction of complex dielectric parameters from the complex reflection coefficient, S11, is based on a full-wave analysis of Maxwell’s equations in cylindrical geometry for semi-infinite samples. The conversion from the reflection coefficient S11 to complex permittivity as a function of frequency was carried out in the DAK software (https://speag.swiss/products/dak/dak-probes/). The influence of possible extra reflection from the sample boundaries of the finite configurations was examined by moving the sample with respect to the open-end coaxial probe. The uncertainty in the S11 measurements was within the range of errors that are acceptable for the setup.

To accurately characterize the dielectric parameters, each tissue sample was subjected to a total of 10 measurements.

When the material interacts with microwaves, two processes occur: energy storage and energy dissipation. Dipoles within the biological sample polarize in reaction to the alternating electric field, leading to energy storage. The dielectric properties of a material are expressed using complex permittivity, as demonstrated in Equation 4 (Martinsen and Heiskanen, 2023).

(4) $$\epsilon^{*}=\epsilon^{\prime }-j\epsilon^{\prime \prime }=\epsilon^{\prime }-j\frac{\sigma }{\epsilon_{0}2\pi f}$$

where the real part *ϵ*′ is the dielectric constant, indicating energy storage. The imaginary part, denoted as ϵ″ is dielectric loss, indicating energy dissipation primarily as heat. σ is the conductivity, reflecting energy loss associated with the dispersion of *ϵ*′. ϵ0 stands for vacuum permittivity.

The experimental data were modeled using a two-pole Cole-Cole equation (Cole and Cole, 1941) (Equation 5) to estimate the dielectric parameter values. When water molecules interact with other biological substances, the spectrum broadens, indicating a distribution of relaxation times. Hence, the Cole-Cole model was selected for its ability to accommodate this distribution when describing the dielectric relaxation of biological materials. Within adipose tissue, the measured dielectric properties are influenced by various molecules: water molecules, protein-bound water, proteins, and neurotransmitters such as noradrenaline (Pethig, 1987; Schwan, 1994; Liewei Sha et al., 2002).

Given the overlap of β- and γ-dispersion within the measurement frequency range (200 MHz – 14 GHz), we selected a two-pole Cole-Cole model. The use of a two-pole formulation is physically justified by the presence of at least two dominant polarization mechanisms within this frequency range, primarily associated with free and bound water fractions exhibiting different molecular mobilities. Water molecules dominate the higher-frequency γ-dispersion region, whereas larger molecules and interfacial polarization mechanisms contribute predominantly to the lower-frequency β-dispersion region. Each mechanism is characterized by a distinct relaxation time reflecting differences in local viscosity, hydrogen bonding, and molecular constraints. The observed deviation from ideal Debye behavior arises from structural and compositional heterogeneity within the tissue, leading to a distribution rather than a single relaxation time. This distribution represents the superposition of multiple microscopic dipolar environments, each contributing differently to the macroscopic dielectric response, and the Cole-Cole broadening parameter therefore provides a phenomenological measure of this heterogeneity and intermolecular interaction within the tissue. Fitting was performed using the Levenberg-Marquardt algorithm based on the non-linear least-squares method. The two-pole Cole-Cole model is expressed as follows:

(5) $$\epsilon^{*}\left(\omega \right)=\epsilon_{\infty }+\frac{\text{Δ}\epsilon_{1}}{1+{\left(j\omega \tau_{1}\right)}^{1-\alpha_{1}}}+\frac{\text{Δ}\epsilon_{2}}{1+{\left(j\omega \tau_{2}\right)}^{1-\alpha_{2}}}$$

where $\text{Δ}\epsilon_{1}=\epsilon_{\text{s}}-\epsilon_{1}$ and $\Delta \epsilon_{2}=\epsilon_{1}-\epsilon_{\infty }$ are the relaxation strength. τ1 and τ2 are the relaxation times. The parameters *ϵ*∞, τ1, τ2, Δϵ1, Δ*ϵ*2, α1, α2 were obtained by fitting the experimental permittivity data to Equation 5.

The percentage of water content was determined by comparing the dielectric loss of deionized water with the measured dielectric loss data of adipose tissue. The water content was calculated by summing over the dielectric loss curve for the measured deionized water and the water contribution of the adipose tissue, as specified in Equation 6:

(6) $$\text{water content}\left[\%\right]=\frac{\sum \left[{\text{ϵ}}^{\prime \prime }\left(\text{water contribution in adipose tissue}\right)\right]}{\sum \left[{\text{ϵ}}^{\prime \prime }\left(\text{deionized water}\right)\right]}\times 100\%$$

All measurements were performed at room temperature (~22 °C) under stable laboratory conditions. Samples were measured within a consistent time window immediately after dissection to minimize temperature-related variability. Although no active temperature-controlled chamber was used, calibration with reference materials and standardized handling procedures ensured measurement consistency across samples.

### 2.5 Near-infrared spectroscopy

NIR spectroscopy measurements were conducted employing Ocean Optics NIRQuest 512 (Ocean Insight, NY, USA). The spectrometer operated in reflectance mode within the wavelength range of 900 to 1700 nm, using a 25 µm slit for an optical resolution of 3.1 nm (full width at half maximum). Connected to the spectrometer was the QP600-2-VIS-NIR fiber (Ocean Insight, NY, USA) with a wavelength range of 400 to 2100 nm.

Before capturing the time series of ex vivo diffuse reflectance data, two spectra were acquired for system calibration. The first spectrum, denoted as the reference spectrum (R(λ)), was measured using a spectralon reflectance standard with high reflectivity, while the light source was active. The second spectrum, the background spectrum [D(λ)], was obtained with the light source turned off. The percentage reflectance as a function of wavelength (T(λ)) was then calculated as follows (Equation 7):

(7) $$\%T\left(\lambda \right)=\frac{S\left(\lambda \right)-D\left(\lambda \right)}{R\left(\lambda \right)-D\left(\lambda \right)}\times 100\%$$

S(λ) represents the sample intensity as a function of wavelength λ. All spectra were obtained using the OceanView software, employing a boxcar smoothing to 5, averaging set to 2 scans, and each scan was conducted with an integration time of 488 ms. To ensure accuracy and minimize drift error, the system underwent a recalibration before each hourly measurement.

### 2.6 Dynamic vapor sorption

The water content of each sample was measured with a Dynamic Vapor Sorption (DVS Intrinsic) instrument from Surface Measurement Systems Ltd., U.K. The samples were automatically weighed in a closed chamber under controlled ambient relative humidity (RH) and temperature. Measurements were performed during the full desorption process at 0% RH and 22 °C to obtain the dry weight of the samples and therefore the initial water content.

### 2.7 Machine learning

Three ML methods were used to classify different types of adipose tissue, i.e., Support vector machine (SVM), Random forest (RF), and eXtreme Gradient Boosting (XGBoost). Stratified 5-fold cross-validation was applied to all the models.

SVM uses a hyperplane to make a separation in a data set to make predictions. The support vectors are data points closest to the hyperplane, which helps to define its position. For non-linear data, the data can be transformed into a higher dimension where it is possible to separate it linearly. The SVM model was built with the Scikit-learn library in Python.

RF is made up of a collection of decision trees. It belongs to the ensemble learning method bagging, which essentially combines multiple individual models that are trained in parallel and combines them into a stronger predictive model. For classification, each tree votes for a class and the majority is the final classification. RF is prone to overfitting as each tree is trained on a random data subset, making it a more generalized model.

XGBoost belongs to the ensemble learning family and works by combining several weak learners to make a strong classifier. It is based on concept boosting, where many weak learners/simple models trained sequentially are combined to make accurate predictions. It iteratively builds a series of decision trees, where each new tree corrects the errors made by the previous ones, consequently minimizing the loss function. XGBoost was run with the XGBoost Python package, and an XGBoost classifier was used for multiclass classification with the softmax objective.

Recursive Feature Elimination (RFE) is a method to remove the least important features. It does this by fitting the model to n features and ranking these features according to importance. Then the least important feature is removed, and the model is fitted again with n − 1 features, and the process is repeated until there are no more features to remove. Since there is no way to know which features are the least important, the model needs to run the fit n times for each cross-validation iteration to know in which order to remove the least important feature, since the features work together and it is not possible to know which features are the least important considered in total (Sanz et al., 2018). RFE with 5-fold cross-validation was used to select the most important frequencies/wavelengths.

Hyperparameter tuning was performed using grid search over predefined parameter ranges. For SVM, the regularization parameter C was varied over a logarithmic range, $C\in \left[10^{-5},\ldots ,10^{5}\right]$. For the Random Forest model, the number of trees was selected from the range n ∈ [50,100,200,300,400,500,600,700,800,900,1000,1500,2000]. For XGBoost, the number of estimators n ∈ [50,100,200,300,500,800,1000], maximum tree depth ∈ [10,50,100,200,300,500,700,1000], and learning rate η ∈ [0.05,0.1,0.2,0.3,1] were explored. Model evaluation was performed using stratified 5-fold cross-validation. Due to this cross-validation scheme, training occurs independently on multiple folds, and therefore a single loss curve is not representative of the overall training behavior. Each model was trained with 200 iterations.

## 3 Results

### 3.1 Morphological difference between iWAT, gWAT, and iBAT is partially reflected by their passive electrical and optical properties

To visualize morphological differences between gWAT, iWAT, and BAT, we performed hematoxylin and eosin staining on paraffin-embedded sections (**Figure 1A**). BAT has the highest density of cells, while gWAT has the lowest cell density as assessed by nuclear staining. Analysis of lipid droplet size shows a similar trend, with BAT mainly harboring small lipid droplets, gWAT large lipid droplets, and iWAT containing a wide variety of lipid droplet sizes (**Figure 1B**). While all three types of adipose tissue have distinct cell sizes and distributions, iWAT and gWAT are morphologically more similar than either is to BAT.

**Figure 1:**
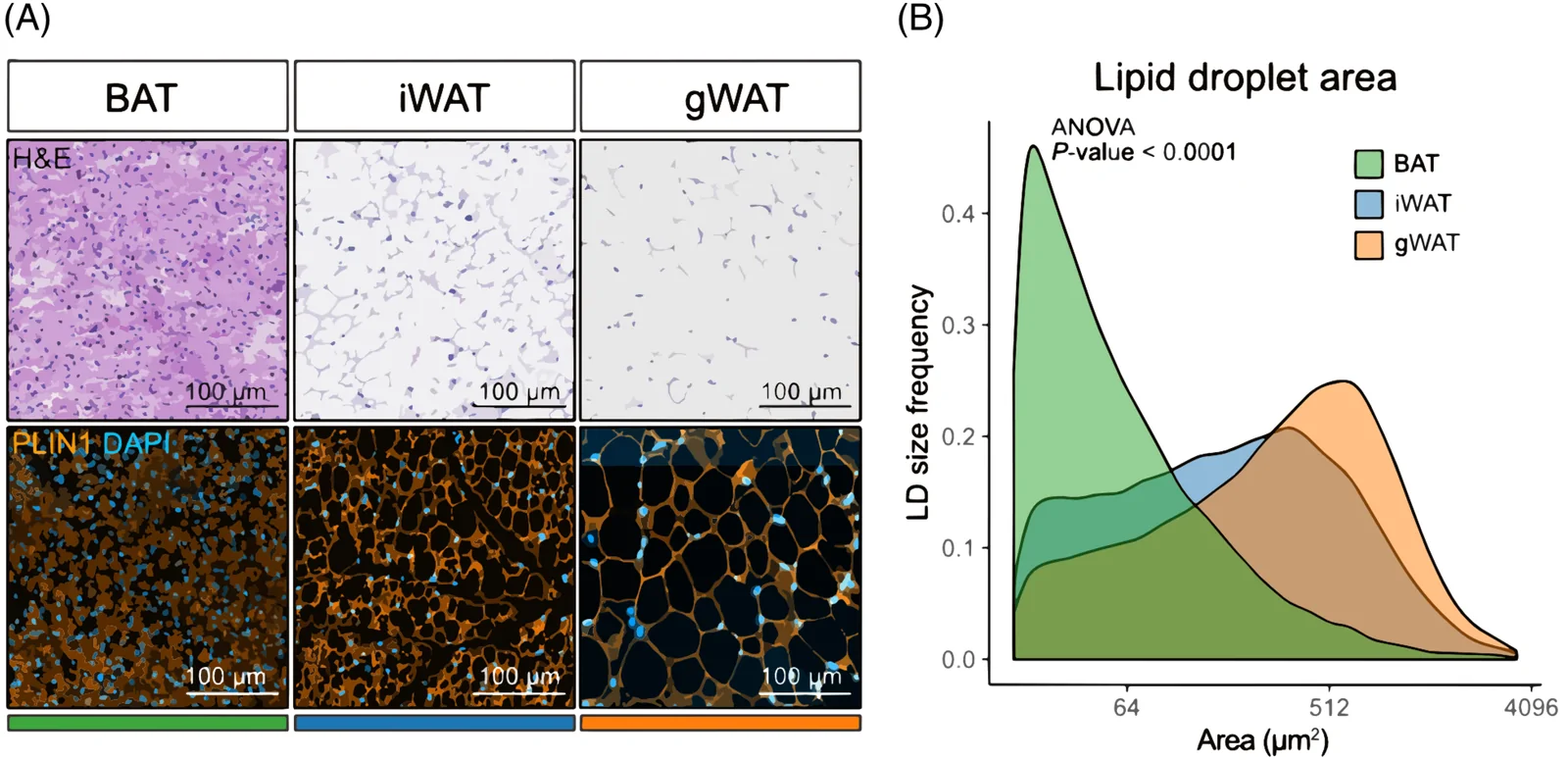
**(A)**. Haematoxylin and eosin staining of inguinal white adipose tissue (iWAT), perigonadal white adipose tissue (gWAT), and brown adipose tissue (BAT). Immunofluorescence staining of lipid droplet marker PLIN1 on iWAT, gWAT, and BAT sections. **(B)**. Quantification of lipid droplets 2D area from PLIN1 staining. n = 7.

To investigate how the morphological differences between adipose depots translated into physical properties, we measured the impedance, dielectric conductivity, and NIR reflectance for all three types of adipose tissue.

Despite a minimal overlap between the impedance of the three types of adipose tissue, BAT and iWAT show a more similar electrical response compared to gWAT (**Figure 2A**). The impedance of gWAT decreases from 25 kΩ at 10 Hz to 16 kΩ at 1 kHz and to 12 kΩ at 100 kHz. The impedance magnitudes of iWAT and BAT also gradually decrease from 16 kΩ and 12 kΩ at 10 Hz to 10 kΩ and 6.5 kΩ at 1 kHz and to 7 kΩ and 5 kΩ at 100 kHz, respectively. Each type of adipose tissue displays the typical behavior of biological tissues. The impedance decreases as the frequency increases due to capacitive cell membranes that behave as short-circuits at higher frequencies (Dean et al., 2008). The EIS dataset presented in **Figure 2A** is an extension of previously published work (Dapsance et al., 2023). It includes data reported in (Dapsance et al., 2023) and additional measurements acquired after its publication. A subset of the newer EIS measurements was performed concurrently with DRS and NIRS on the same samples in the later stages of the study.

**Figure 2:**
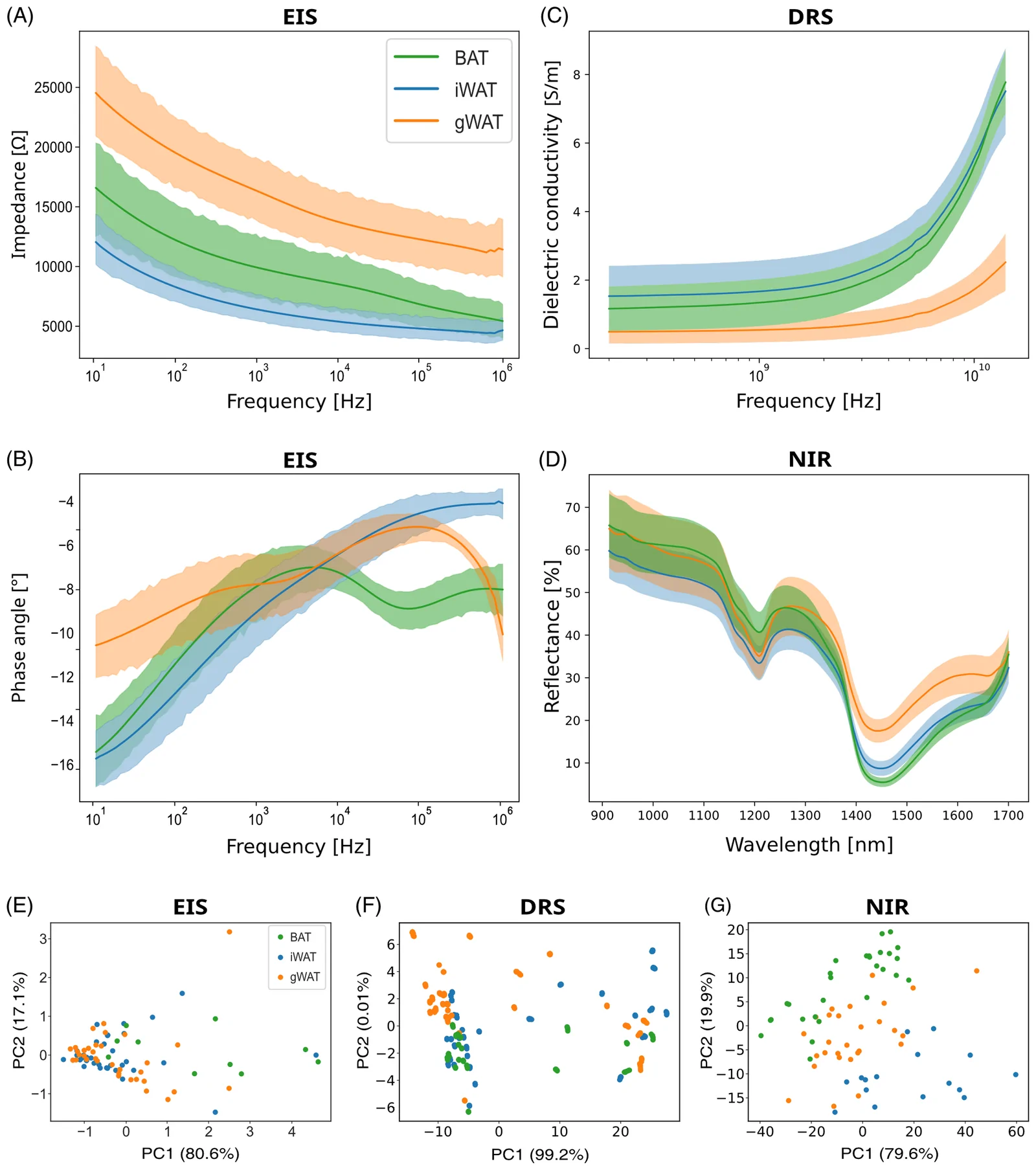
Electrical impedance (EIS), dielectrical relaxation (DRS), and near-infrared (NIR) spectroscopy plots for brown (BAT), inguinal white (iWAT), and perigonadal adipose tissue (gWAT) with 95% confidence intervals. **(A)** Impedance of BAT (n = 11), iWAT (n = 36) and gWAT (n = 34) from 10 Hz to 1 MHz. **(B)** Phase angle of BAT (n = 11), iWAT (n = 38) and gWAT (n = 37) from 10 Hz to 1 MHz. **(C)** Dielectric conductivity of BAT (n=18), iWAT (n=31), and gWAT (n=32) from 200 MHz to 14 GHz. **(D)** NIR Reflectance of BAT (n = 16), iWAT (n = 28) and gWAT (n = 28) from 900 nm to 1700 nm. Principal component analysis for **(E)** electrical impedance spectroscopy measurements of BAT, iWAT, and gWAT, **(F)** dielectric relaxation spectroscopy measurements of BAT, iWAT, and gWAT, and **(G)** near-infrared spectroscopy measurements of BAT, iWAT, and gWAT.

BAT and iWAT share a similar trend in phase angle at low-frequencies, -16° to -7° between 10 Hz and 8 kHz, while it evolves from -10.5° to -7°for gWAT (**Figure 2B**). In the medium-to-high frequency range, between 5 kHz and 50 kHz, the phase angles of gWAT and iWAT are almost identical, -7° to -5°, while the phase angle of BAT drops to -8.5°. From 50 kHz, the phase angles of BAT and iWAT stabilize around -8,5° and -4° respectively, while the phase angle of gWAT falls to -10° at 1 MHz. This means that at low frequencies, the measurements on iWAT and BAT are more dominated by interface polarization and the effect of cell membranes than on gWAT. In the medium frequencies, iWAT and gWAT become mostly resistive, while BAT maintains a higher capacitive contribution. At high frequencies, only gWAT exhibits a variation towards capacitive processes.

The dielectric conductivity behavior for iWAT and BAT overlaps, while gWAT stands out with a lower conductivity, across the whole frequency range (**Figure 2C**). The dielectric conductivity of both BAT and iWAT remains consistent to 1.2 S/m and 1.5 S/m, respectively, from 200 MHz to 1 GHz. The measurements on gWAT are steady around 0,5 S/m up to 2–3 GHz, before gradually rising to 2,5 at 14 GHz. BAT and iWAT follow an increasing trend in conductivity to 3 S/m from 1 GHz to 5 GHz before a steeper rise to 7,5 S/m at 14 GHz. The conductivity of gWAT is consistent with previous reports on adipose tissue, while the conductivity of iWAT and BAT is closer to higher water tissues such as muscle or liver (Gabriel et al., 1996). Interestingly, a crossover between the dielectric conductivity curves of iWAT and BAT is observed near ~10 GHz. This behavior likely reflects differences in high-frequency dielectric relaxation mechanisms, particularly those associated with free water molecules (γ-dispersion). At these frequencies, dielectric properties are dominated by the dynamics of mobile water, and the crossover may indicate differences in the relative contributions of free and bound water fractions, as well as variations in cellular density and intracellular structure between tissues.

On the NIR reflectance spectrum, all three types of adipose tissue overlap between wavelength 900–1360 nm and show a clear absorption peak around 1200 nm that is slightly less sharp for BAT, 41% compared to 34% and 35% for iWAT and gWAT, respectively (**Figure 2D**). gWAT starts to deviate from iWAT and BAT from around 1360 nm and reaches an 18% reflectance trough at 1440 nm while iWAT and BAT reach their lowest reflectance of 8.7% and 4.9%, respectively. The distinct peaks at 1200 nm and 1440 nm correspond to the absorption of lipids and water, respectively (Wilson et al., 2015).

Among the three measurement techniques, gWAT shows the highest impedance, lowest capacitive contribution, lowest conductivity, and weakest water absorption peak. Despite slight differences in impedance and capacitive contribution at higher frequencies, BAT and iWAT display highly similar conductivity and water absorption peak. To further assess how each method performs in discriminating between BAT, iWAT, and gWAT adipose tissue, we performed a principal component analysis (PCA) (**Figures 2D–F**).

For EIS measurements from 10 Hz to 1 MHz, the first principal component (PC1) explains 80.6% of the variation while the second component (PC2) explains 17.1% of the variation (**Figure 2D**). However, no group clearly stands out.

For DRS measurements from 200 MHz to 14 GHz, the PC1 and PC2 explain 99.2% and 0.01% of the variation, respectively (**Figure 2E**). The spread of data points is lower than for EIS, but remains uneven.

For NIR spectroscopy measurements from 900 to 1700 nm, the PC1 and PC2 explain 79.6% and 19.95% of the variation, respectively (**Figure 2G**). The data points representing the three groups are notably separated, indicating larger variations between the groups from the NIR spectroscopic measurements than with EIS and DRS. This suggests that NIRS is sensitive to differentiating the three types of tissue.

All three measurement techniques reveal tissue differences, and each technique is affected by various biophysical properties. EIS measurements are dominated by tissue geometry and bulk conductivity. The PCA outputs clusters that overlap due to the moderate contribution of the type of tissue to the variance. Specifically, the variance in conductivity from the DRS measurements is almost entirely explained by a single component, which is likely to be the water content of the tissue. The PCA on the reflectance from NIRS shows a rather clear clustering along the two principal components, which suggests that two tissue properties, e.g., water content and lipid content, are independently captured by NIRS and align with the different types of tissue.

### 3.2 DRS accurately discriminates adipose tissue depots

Despite observable differences between each type of adipose tissue in impedance, phase angle, dielectric conductivity, and reflectance, the PCA failed to reveal a clear distinction between the groups for EIS and DRS. As a consequence, three ML models, SVM, RF, and XGBoost, were tested for automatic classification of adipose tissue types.The dataset consisted of three classes corresponding to BAT, iWAT, and gWAT samples. Stratified 5-fold cross-validation was employed to ensure that each fold preserved the class distribution of the full dataset. Model evaluation was performed using the cross_validate function from Scikit-learn. The scoring metric used was accuracy, defined as the proportion of correctly classified samples over the total number of samples. The reported performance corresponds to the mean accuracy and standard deviation across the five folds.

The performance of each model on multiple parameters is summarized in **Table 1**. For EIS measurements, the reactance parameter shows a slightly higher accuracy than that of the resistance parameter – in all three models – but the variation in accuracy is between 8–12%. The SVM model resulted in the best accuracy, with 85.15 ± 8.53% for the reactance and 81.47 ± 10.47% for the resistance. For permittivity data, the dielectric constant data markedly outperformed the dielectric loss data for all three models. All three models showed an accuracy above 98% for the dielectric constant, with a slight edge with the RF model, where it scored 99.75 ± 0.3%. For NIR reflectance, the SVM model achieved better accuracy than the other models, with 83.43 ± 15.13%, although the standard deviation is the largest among all parameters tested.

**Table 1:** Model performance for classifying BAT, iWAT, and gWAT adipose tissue types.

Methods	EIS		Permittivity		NIR
	*R*	*X*	*ε’*	*ε”*	*Reflectance*
SVM	81.47 ± 10.47	85.15 ± 8.53	98.4 ± 1.0	39.51 ± 0.17	83.43 ± 15.13
RF	59.19 ± 10.35	75.37 ± 8.61	99.75 ± 0.3	38.52 ± 4.53	70.57 ± 17.72
XGBoost	64.26 ± 10.44	77.79 ± 12.16	99.26 ± 0.6	39.14 ± 5.43	76.29 ± 11.65

Results from 5-fold cross-validation show the mean accuracy and standard deviation. (EIS: Electrical Impedance Spectroscopy; SVM: Support Vector Machine; RF: Random Forest; XGBoost: eXtreme Gradient Boosting).

Based on the classification results, the best-performing model for each data type was selected to investigate the most important frequencies or wavelengths for adipose tissue classification, i.e., the RF model for DRS dielectric constant measurements and SVM models for EIS reactance data and NIR spectroscopy reflectance data. The results from RFE (**Figure 3**) show how the mean test accuracy changes with the number of features. For all parameters, the highest accuracy is reached without including all features in the model. For the reactance in EIS (**Figure 3A**), the accuracy slightly decreases when including more than 11 features. For the dielectric constant parameter from DRS (**Figure 3B**), the test accuracy gradually increases until a plateau is reached around 80 features included in the model. For NIR reflectance (**Figure 3C**), the mean test accuracy peaks for around 195 features and remains stable even with more features.

**Figure 3:**
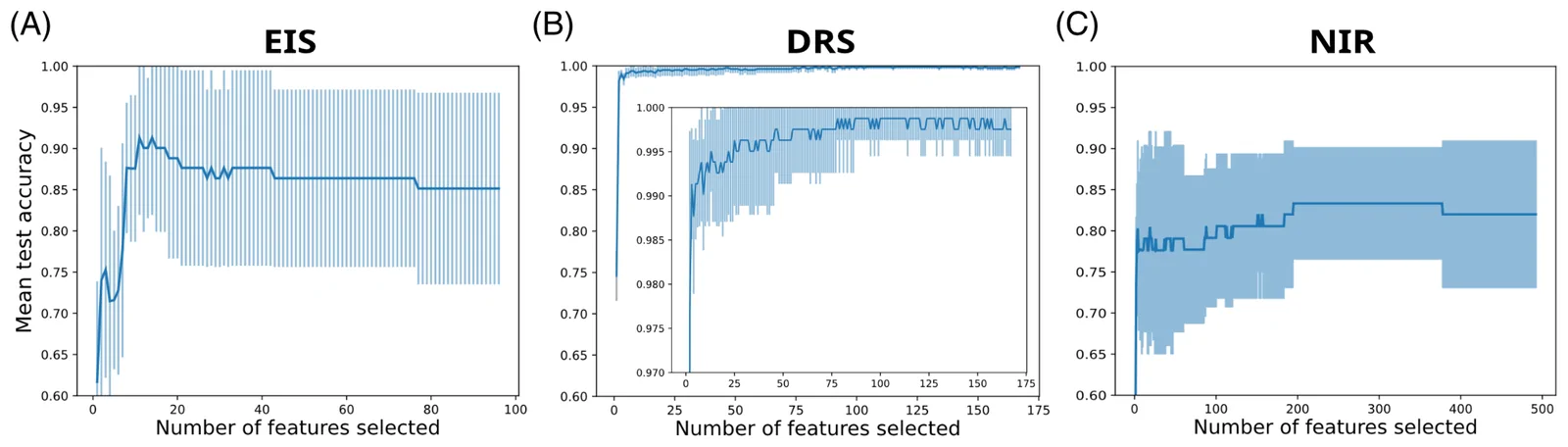
Number of selected features of **(A)** the reactance parameter from electrical impedance spectroscopy, **(B)** the dielectric constant parameter, and **(C)** the NIR reflectance parameter versus the mean test accuracy from recursive feature elimination with 5-fold cross-validation.

To assess the reliability of the best performing model for each method with a limited number of features, the mean accuracy obtained with only the top 10 features is compared to the maximal mean accuracy (**Table 2**). For the reactance parameter, 11 out of 96 frequencies between 10 Hz and 1 MHz are selected by the SVM model. The accuracy slightly decreases from 87.65% to 82.79% when using 11 to 10 frequencies, respectively. For the dielectric constant, 80 out of 167 frequencies between 200 MHz and 14 GHz are kept by the RF model. When using only 10 frequencies, the accuracy drops from 99.88% to 86.54%. For the reflectance parameter, the SVM model identifies 195 out of 512 wavelengths between 900 and 1700 nm. Restricting the model to the 10 best features brings the accuracy from 80.57% down to 51.14%.

**Table 2:** Model performance with RFE selected features for reactance (EIS), dielectric constant (DRS), and reflectance (NIR). RF model was used for Dielectric constant data, and SVM was used for both reactance and reflectance data.

Model performance	EIS		DRS		NIR	
Total #features	96		167		512	
Selected #features	11	10	80	10	195	10
Accuracy (%)	87.65	82.79	99.88	86.54	80.57	51.14
Standard deviation	7.91	8.96	0.25	2.51	12.92	10.85

In summary, the classification of different types of adipose tissue reached good accuracy (≈80%) from EIS and NIRS measurements with SVM. The accuracy was maintained for EIS even with 10 or 11 features. In addition, DRS measurements combined with SVM, RF, or XGBoost algorithms could discriminate between iWAT, gWAT, and BAT with excellent accuracy (>99%) using 80 different frequencies and even with good accuracy with only 10 frequencies.

### 3.3 DRS is a reliable method to dynamically estimate water content in adipose tissue

The comparative results obtained from multiple measurement techniques presented in the previous sections suggest clear variations between iWAT, gWAT, and BAT in their passive electrical and optical properties. In particular, the automatic classification of the three adipose tissue types from DRS performs better than EIS and NIRS. This may seem counterintuitive given the facts that (i) electrical conductivity is highly dependent on the amount of water in the sample and that (ii) adipose tissue is predominantly composed of electrically insulating lipid molecules and cell membranes. However, iWAT, gWAT, and BAT exhibit distinct cell size, distribution, and lipid content as suggested here, very different water content. This difference is particularly notable around 1440 nm in the reflectance spectrum (**Figure 2D**), where water is known to show an absorption peak.

To confirm the estimation of water content, samples from each adipose tissue depot were first measured with DRS, and then with the gold-standard DVS method. The estimated water content obtained from DRS showed strong linear agreement with the actual water content measured by DVS (R2 ≈ 0.97). Pearson correlation analysis confirmed a very strong positive relationship between the two methods (r ≈ 0.98, p < 0.001), indicating that the DRS estimates reliably track the DVS measurements across the investigated samples (**Figure 4A**). Apart from a single outlier around 15%, gWAT has a 5% water content, while iWAT and BAT exhibit a water content around 13–17% and 25–30%, respectively. Additionally, the dielectric loss calculated from Cole-Cole modeling of DRS measurements can be used to estimate the water content of adipose tissue samples. As shown in **Figure 4B**), both iWAT and BAT have a water content of approximately 20–40%, while it ranges between 3 and 12% for gWAT.

**Figure 4:**
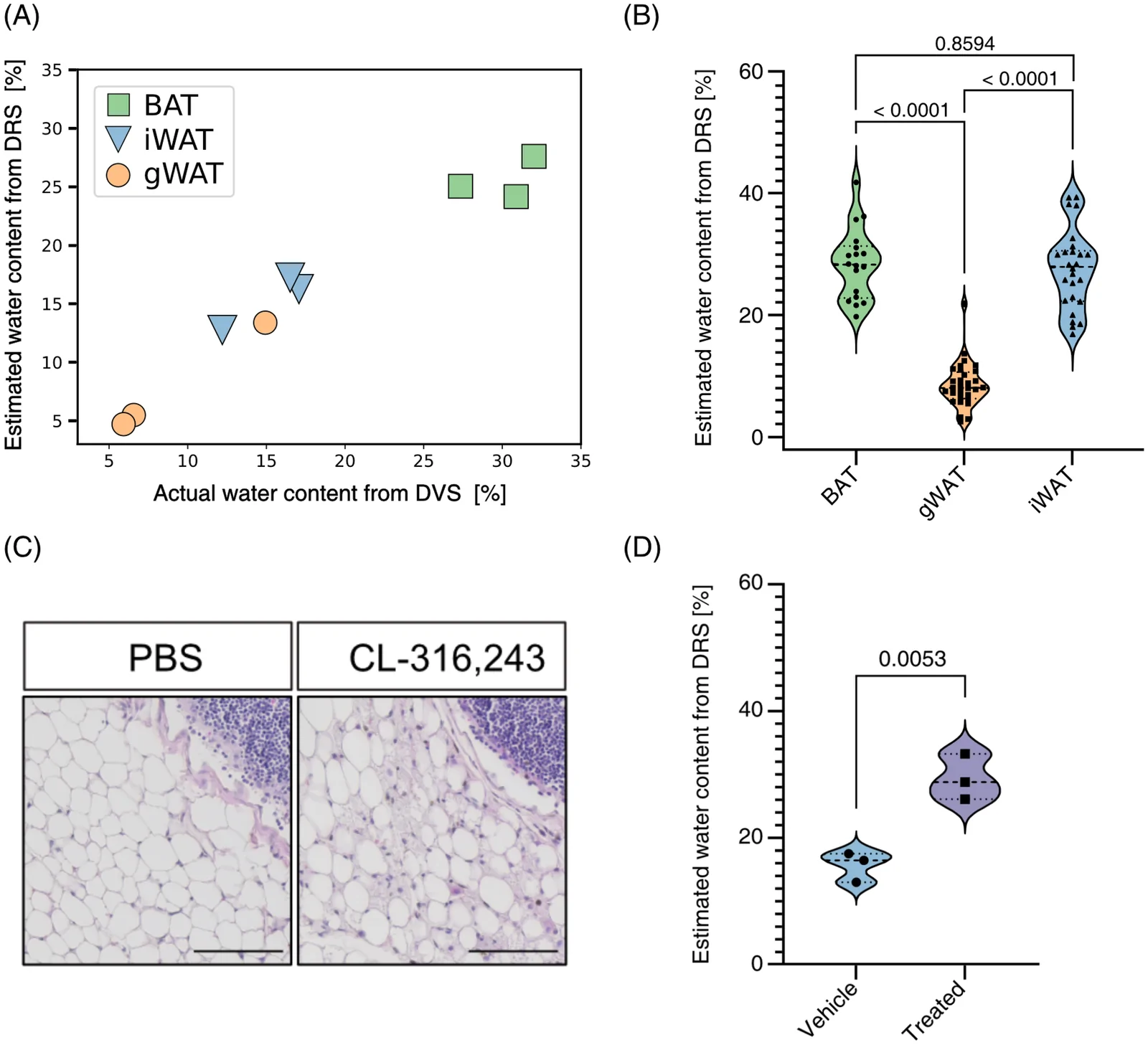
**(A)** Correlation between water content measured from the dynamic vapor sorption (DVS) instrument and water content estimated from the dielectric relaxation spectroscopy (DRS) method. (n=3) **(B)** Violin plot comparing the distribution of estimated water content among BAT (n=18), gWAT (n=26), and iWAT (n=26) using one-way ANOVA, followed by Tukey’s multiple-comparisons test. **(C)** Hematoxylin eosin staining of iWAT from mice treated with PBS or CL-316,243 for 2 days. Scale: 100 μm. **(D)** Violin plot comparing the distribution of estimated water content between iWAT of mice treated with CL-316,243 (n=3) or vehicle (n=3) using a two-sided unpaired Welch’s t-test. Each point represents an individual mouse tissue sample.

In our housing conditions, iWAT contains predominantly white and some beige adipocytes. However, white adipocytes can transdifferentiate into beige adipocytes following cold exposure, in response to the activation of the sympathetic nervous system (Shao et al., 2019). To test whether the water content is affected by the proportion of beige adipocytes, we induced iWAT beiging by treating mice with a sympathomimetic adrenergic receptor agonist CL-316,243 for 2 days. As expected, this treatment leads to the appearance of beige adipocytes, characterized by their eosinophilic cytoplasm (**Figure 4C**). In line with the higher water content in BAT and iWAT as compared to gWAT, iWAT beiging following CL-316,243 treatment correlates with an increase in water content from 18 to 30% (**Figure 4D**). Overall, these results show that DRS can accurately estimate the water content, providing a reliable and non-destructive method to assess the beiging of white adipose tissue.

## 4 Discussion

This work compared how EIS, DRS, and NIRS enable the characterization and classification of adipose tissue, here determining the passive electrical and optical properties of different mouse adipose tissue depots (BAT, iWAT, and gWAT), using histology as structural reference. Histological analysis indicated structural similarity between iWAT and gWAT, both harboring large unilocular lipid droplets. However, EIS, DRS, and NIRS measurements consistently revealed a higher similarity between BAT and iWAT, with gWAT showing distinct features. This apparent discrepancy reflects the complementary nature of the methods: histology captures macroscopic morphology, while electrical and optical techniques probe molecular composition, membrane density, and water content.

EIS measurements showed that BAT and iWAT have a lower impedance than gWAT across the full frequency range, consistent with a higher water content and cellular density. The higher resistance in BAT compared to iWAT suggests a lower overall ionic conductivity due to a higher density of small cells, and the differences in reactance at higher frequencies reflect the size of lipid droplets and therefore the intracellular conductivity. These differences can be interpreted in light of known biological variations between adipose depots. BAT is characterized by smaller adipocytes, high mitochondrial density, and increased vascularization, all of which contribute to higher ionic conductivity and stronger capacitive effects. In contrast, gWAT contains larger unilocular adipocytes with lower intracellular water content, resulting in reduced conductivity and weaker membrane-related polarization effects. iWAT exhibits intermediate characteristics due to the presence of both white and beige adipocytes, which likely explains its partial overlap with BAT in electrical and dielectric behavior.

The two-electrode setup may lead to electrode polarization effects at low frequencies. However, the use of relatively large Ag/AgCl electrodes and the consistently low phase angles observed across the measured frequency range indicate that electrode polarization impedance contributes only minimally to the measured data. The predominantly conductive behavior of the tissue further suggests that the recorded impedance is governed primarily by the intrinsic electrical properties of the adipose tissue rather than by the electrode–tissue interface. This interpretation is consistent with our previous estimation of the polarization impedance, which suggested that the total impedance is not dominated by capacitive effects (Dapsance et al., 2023). In addition, the effect of electrode polarization largely dissipates above 1 kHz, where the distinction between the various types of adipose tissues remains as clear as at lower frequencies.

An important limitation of the present EIS measurements is that the recorded impedance represents a two-electrode measurement and may be influenced by sample geometry and electrode–tissue contact conditions. We used fixed electrodes, standardized handling procedures and selected samples of comparable size. However, tissue thickness, volume and contact area were not systematically quantified nor used for normalization. Consequently, differences in impedance magnitude between depots may reflect not only intrinsic differences in tissue composition, including water content, cellularity, membrane-related polarization, and lipid-droplet structure, but also differences in sample thickness, current-path length, contact area, and compression at the electrode interface. Therefore, the EIS results should be interpreted as differences in the overall electrical response of the tissue samples, which may reflect both tissue composition and differences in sample size, thickness, or electrode contact. Future studies should incorporate controlled sample thickness and compression, systematic measurement of sample geometry, normalization to tissue volume or mass to disentangle these contributions.

DRS measurements showed distinctly reduced dielectric conductivity in gWAT, while the spectra of BAT and iWAT largely overlapped, indicating comparable abilities to store and dissipate electromagnetic energy. The rapid increase in conductivity for iWAT and BAT in the GHz range corresponds to a higher presence of mobile water compared to gWAT, considering its modest increase in conductivity (Schwan and Foster, 1977). The crossover observed between iWAT and BAT at high frequencies further suggests that subtle differences in water organization and molecular mobility exist between these tissues. Such effects are not captured by low-frequency electrical measurements or histology, highlighting the added value of broadband dielectric spectroscopy for probing tissue composition at the molecular level.

NIR spectroscopy further highlighted differences in tissue composition, particularly at the water absorption peak around 1440 nm. gWAT exhibited markedly lower water absorption than iWAT and BAT, according to its high lipid content and low intracellular and extracellular conductivity. The lowest absorption peak at 1200 nm is consistent with the lower lipid density in BAT. These spectral differences therefore support the ability of NIR spectroscopy to distinguish adipose tissue depots based on their relative lipid and water composition, suggesting its potential as a complementary tool for identifying metabolic remodeling such as adipose tissue beiging.

In addition to cellular composition, the extracellular matrix (ECM) may contribute to the measured electrical and optical properties. Variations in collagen content, fibrosis, and ECM remodeling can alter tissue conductivity, dielectric dispersion, and light scattering. Increased fibrosis, for example, is expected to reduce water mobility and increase structural heterogeneity, thereby influencing both impedance and dielectric relaxation behavior. Similarly, ECM organization may affect optical scattering in NIR measurements. Although ECM properties were not directly assessed in this study, these factors could provide an additional layer of contrast and may enable indirect assessment of tissue stiffness or fibrosis in future applications.

From a practical perspective, EIS offers advantages in terms of cost, simplicity, and ease of calibration, whereas DRS provides rapid measurements but requires specialized dielectric materials. NIRS is the fastest and simplest method to deploy, yet its limited sampling volume and sensitivity to probe placement—particularly in heterogeneous tissues such as iWAT introduce variability. Although precautions were taken to avoid direct measurement over lymph nodes, tissue heterogeneity remains a significant source of uncertainty.

Importantly, none of these techniques should be considered universally optimal in isolation. Instead, they provide complementary perspectives on adipose tissue biology. EIS primarily reflects bulk electrical properties and membrane-related effects, DRS is highly sensitive to molecular polarization and water content, and NIRS captures optical signatures related to lipid–water composition. While DRS achieved the highest classification accuracy in this study, EIS and NIRS offer practical advantages in terms of cost, simplicity, and ease of implementation.

The combined use of these techniques therefore represents a powerful multimodal approach, enabling a more comprehensive characterization of adipose tissue than any single method alone. Such integration is particularly relevant for heterogeneous tissues like iWAT, where structural, compositional, and functional properties may diverge.

The indirect nature of EIS, DRS, and NIRS creates a need for further modeling to relate signals to biological parameters. In addition, the complexity of adipose tissues, including their anisotropic cell distribution, rich intracellular spaces, and heterogeneous water distribution, can hardly be characterized by these methods. Although equivalent circuit modeling and parameter extraction from the Cole equation are the most standard methods to analyze EIS measurements on biological tissue, PCA was suggested to perform better for a classification task (Nejadgholi and Bolic, 2015). In this work, PCA failed to extract identifiable features from EIS and DRS measurements based on the type of adipose tissue. Therefore, we compared ML algorithms, SVM, RF, and XGBoost, to classify the types of adipose tissue based on the EIS, DRS, and NIRS measurements. DRS measurements resulted in the highest accuracy (>99%) with the three ML methods, although the performance was constrained by the size of the data set. The limited number of samples precluded the use of more complex models, such as neural networks and reduced confidence in generalization beyond the current cohort. RFE was further implemented to identify a small number of informative frequencies and wavelengths, suggesting that simplified and application-specific instrumentation could be developed.

EIS, DRS, and NIRS are highly sensitive to the amount of water in the tissue. More precisely, the dielectric properties extracted from DRS measurements are closely related to the water content (Rodrigues et al., 2016). The water content in adipose tissue is inversely correlated with lipid accumulation and reflects the proportion of beige or brown adipocytes. That is why in this work, the water content of BAT, iWAT, and gWAT was inferred from DRS measurements after preliminary validation against DVS. A previous study also reported a water content of approximately 10% and 20% in rat gWAT and iWAT, respectively (Reinoso et al., 1997). Furthermore, DRS was shown to be effective in measuring the increased water content caused by the increased density of beige adipocytes in iWAT from mice treated with CL-316,243. This work thus confirms the correlation between beiging and the water content of adipose tissue. Other studies have used advanced optical and imaging techniques to quantify adipose tissue beiging in mice. Dinish et al. used diffuse reflectance spectroscopy and multispectral imaging to exhibit the beiging process of iWAT ex vivo from mice treated with CL-316,243 (Dinish et al., 2017). The authors validated the gradual change in reflectance and image intensity 4 and 7 days after treatment, compared to control iWAT and BAT, with UCP1 and PGC-1α expression and histology. Chan et al. combined multispectral imaging with ultrasound tomography for the same purpose in living mice (Chan et al., 2018). The authors assessed iWAT beiging over 10 days in transgenic mice by quantifying the near-infrared fluorescent activity corresponding to UCP1 expression. A more recent review on optical imaging for brown and beige adipose tissue highlights its potential for assessing brown and beige adipose tissue activation, while noting limitations related to imaging depth, cost, and instrumentation complexity (Liu et al., 2024). In contrast to these imaging-based approaches, the present work demonstrates that dielectric spectroscopy can provide a rapid, label-free, and quantitative assessment of adipose tissue composition and beiging by exploiting differences in tissue water content and dielectric properties.

Despite these advantages, several practical challenges remain when translating such techniques to routine or longitudinal monitoring. Current methods for monitoring beiging in vivo require expensive instrumentation and complex procedures. The setup for ex vivo assessment of beiging with optical or dielectric spectroscopy remains costly and poorly compatible with longitudinal monitoring. Several limitations of the present study should be emphasized. First, the absence of perfusion, physiological temperature regulation, and metabolic activity can affect electrical and dielectric properties, limiting direct translation to in vivo settings. Second, although measurements were performed under stable room temperature conditions (~22 °C), no active temperature regulation system was used. Given the known sensitivity of dielectric properties to temperature, particularly in water-containing tissues, small variations may influence absolute values. However, consistent handling, timing, and calibration procedures across all samples ensure that comparative trends between tissue types remain reliable. Finally, EIS, DRS, and NIRS provide indirect measurements that require further processing, modeling, and analysis, which can introduce uncertainties in the estimations.

Despite these limitations, the complementary information provided by EIS, DRS, and NIR spectroscopy demonstrates their potential for label-free, fast, and non-destructive characterization of adipose tissue. Future advances in probe design, miniaturization, and calibration, combined with larger datasets and in vivo validation, will be required to fully establish these methods as reliable tools for monitoring adipose tissue remodeling and beiging – crucial adaptive transformation of the adipose tissue in response to stresses and aging.

## 5 Conclusion

This study reveals how EIS, DRS, and NIR spectroscopy can be used to characterize adipose tissue types in a faster and non-destructive manner compared to standard histological methods. All three methods showed that iWAT and BAT are similar in both the passive electric and optical reflectance properties. That contrasted with histological results showing that iWAT and gWAT share similar morphological properties. Given the strong relationship between the water content of tissue, its measured dielectric and absorbance properties, and its biological features, a non-invasive technique to assess the water content of a tissue offers a wide range of research and clinical applications. DRS can then provide an estimate of the beiging process in iWAT based on the water content. Beiging is a hallmark of cold resistance and metabolic adaptation, but methods for the longitudinal study of this process are lacking. Its effect on energy expenditure explains the clinical interest in this process as a potential treatment for obesity (Harms and Seale, 2013).

## Data Availability

The raw data supporting the conclusions of this article will be made available by the authors, without undue reservation.
