# Supplementary material for: Determination of adipose tissue beiging from passive electrical and optical properties
Source: Front Physiol. 2026 Oct 29;17:1917194. doi: 10.3389/fphys.2026.1917194 (PMC13754682; doi:10.3389/fphys.2026.1917194)
Supplement: Supplementary file 1 [file DataSheet1.pdf]

frontiers

# Supplementary Material

## 1 SUPPLEMENTARY FIGURES

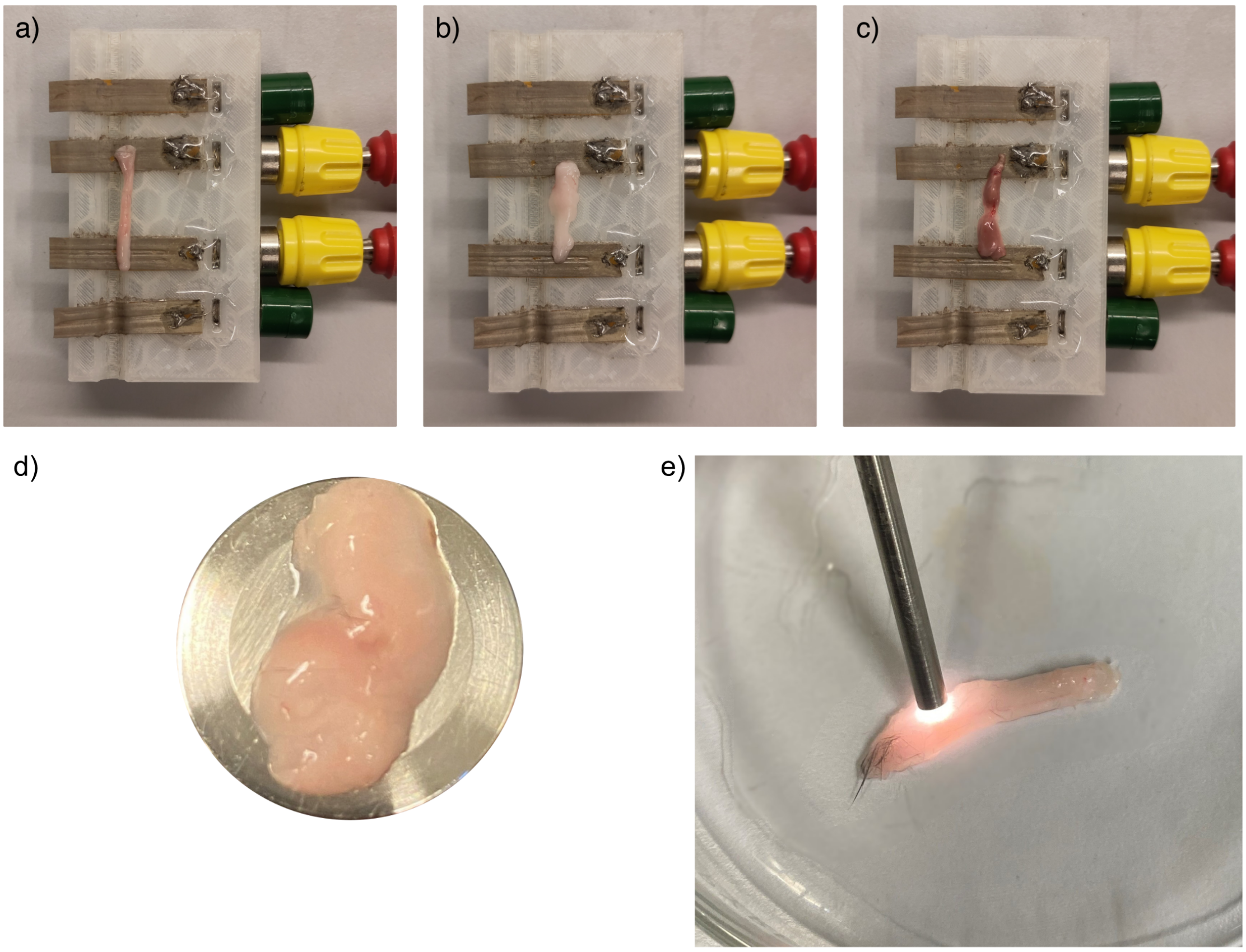

**Supplementary figure S1.** Experimental setup for *ex vivo* bioimpedance measurements of mouse adipose tissue on a custom platform. a) inguinal white adipose tissue (iWAT). b) perigonadal white adipose tissue. c) brown adipose tissue (BAT). c) Dielectric relaxation spectroscopy experimental setup for *ex vivo* measurements of mouse adipose tissue with the DAK 3.5 probe. d) Near-infrared spectroscopy experimental setup for *ex vivo* measurements of mouse adipose tissue with the VIS-NIR optical fiber.

| Method | iWAT | gWAT | BAT |
|---|---|---|---|
| EIS | 36 | 34 | 11 |
| DRS | 31 | 32 | 18 |
| NIR | 16 | 28 | 28 |

**Table S1.** Number of samples per adipose tissue class for each measurement modality.

“‘

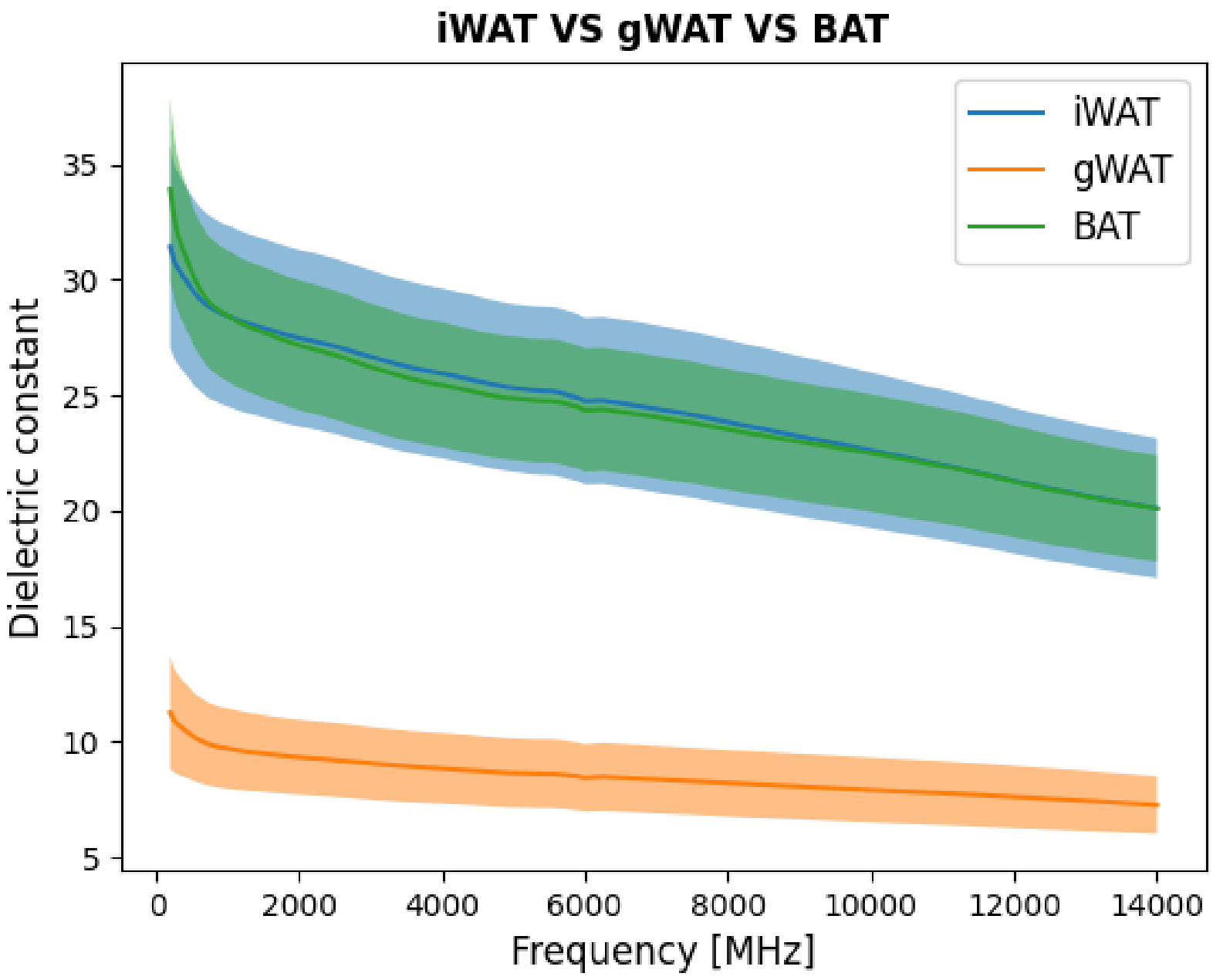


**Supplementary figure S2.** dielectric constant ($\epsilon'$) of BAT, iWAT, and gWAT from 200 MHz to 14 GHz with 95% confidence intervals

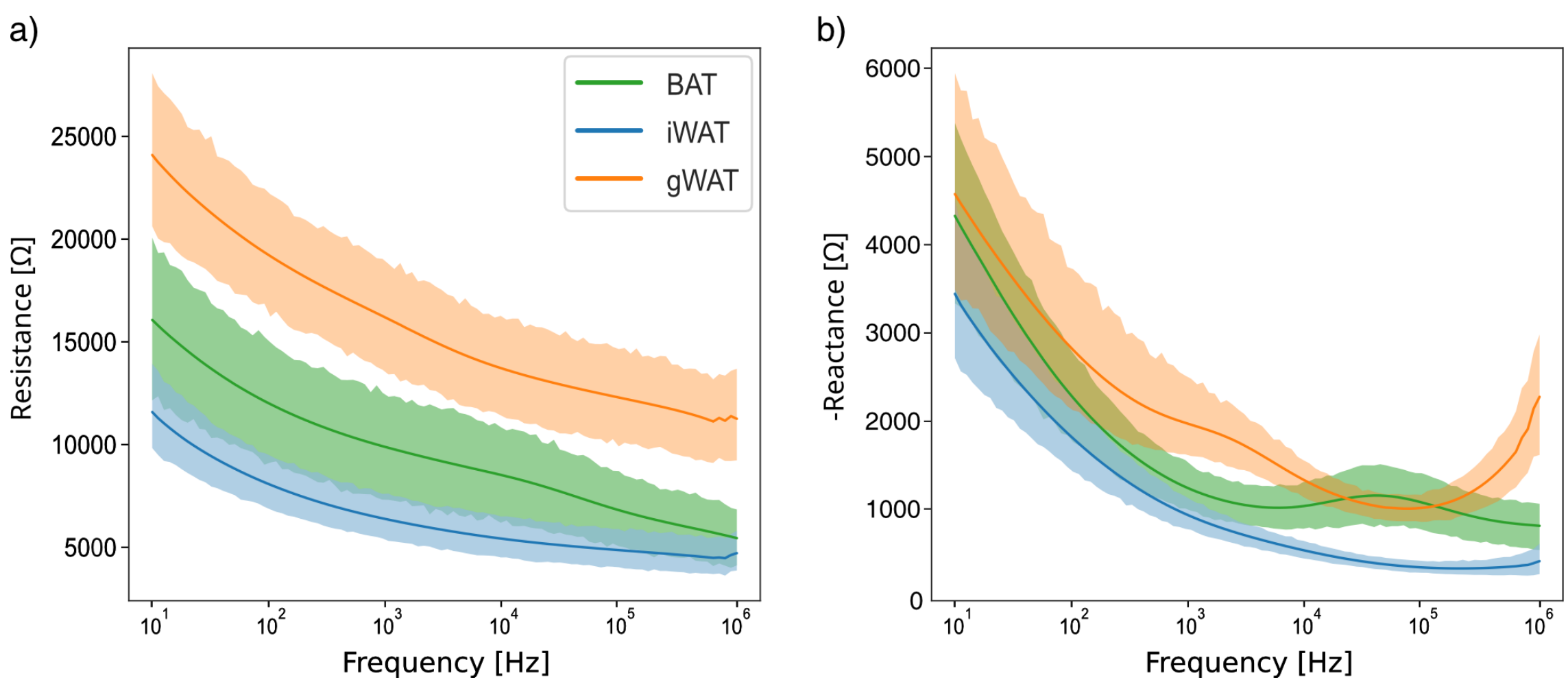


**Supplementary figure S3.** Electrical impedance spectroscopy (EIS) from 10 Hz to 1 MHz with 95% confidence intervals a) Resistance, b) Absolute reactance of BAT (n = 11), iWAT (n = 38) and gWAT (n = 37).

**Table S2.** Random Forest Confusion Matrix

| True / Predicted | iWAT | gWAT | BAT |
|---|---|---|---|
| iWAT | 28 | 7 | 1 |
| gWAT | 7 | 25 | 2 |
| BAT | 4 | 6 | 1 |

**Table S3.** SVM Confusion Matrix

| True / Predicted | iWAT | gWAT | BAT |
|---|---|---|---|
| iWAT | 36 | 0 | 0 |
| gWAT | 6 | 27 | 1 |
| BAT | 3 | 1 | 7 |

**Table S4.** XGBoost Confusion Matrix

| True / Predicted | iWAT | gWAT | BAT |
|---|---|---|---|
| iWAT | 29 | 6 | 1 |
| gWAT | 5 | 25 | 4 |
| BAT | 4 | 4 | 3 |

**Table S5.** Classification Performance Metrics

| Model | F1 Macro (Mean ± Std) | Recall Macro (Mean ± Std) | Specificity (Mean ± Std) |
|---|---|---|---|
| Random Forest | $0.5236 \pm 0.0928$ | $0.5361 \pm 0.0877$ | $0.8120 \pm 0.1034$ |
| SVM | $0.8293 \pm 0.1550$ | $0.8159 \pm 0.1563$ | $0.9215 \pm 0.0859$ |
| XGBoost | $0.6135 \pm 0.1511$ | $0.6107 \pm 0.1380$ | $0.8381 \pm 0.0590$ |
